# Supplementary material for: Distributed context-dependent choice information in mouse posterior cortex
Source: Nat Commun. 2023 Jan 12;14:192. doi: 10.1038/s41467-023-35824-6 (PMC9837177; doi:10.1038/s41467-023-35824-6)
Supplement: Supplementary file 1 — Supplementary Information [file 41467_2023_35824_MOESM1_ESM.pdf]

Supplementary Figures

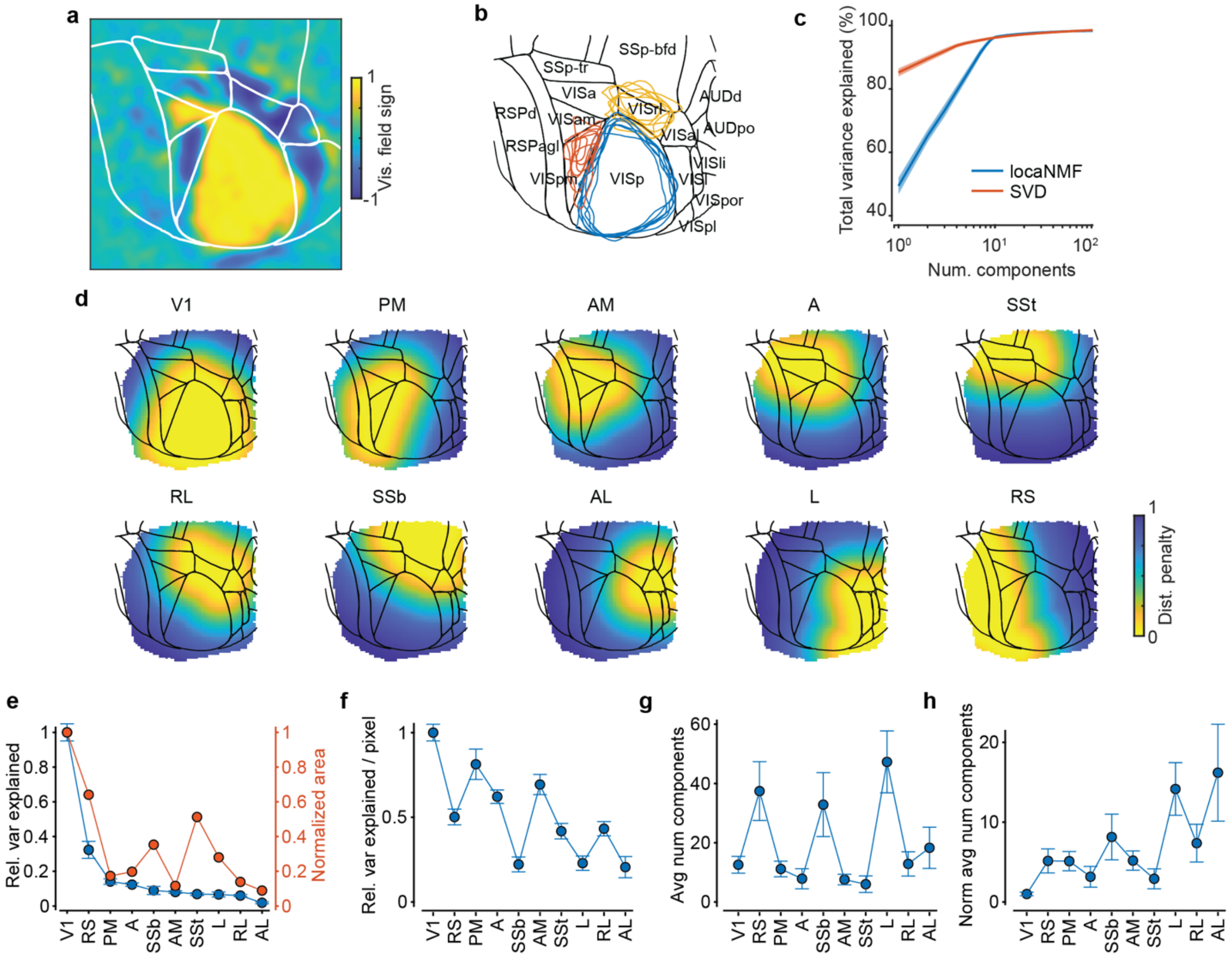

Supplementary Figure 1 | a, Left: Representative example of the visual field sign from a retinotopy experiment used to identify visual areas. Right: Superposition of retinotopically aligned animals (blue, red, and yellow contours) with the Allen Brain Atlas. **b,** Pixel-wise distance penalty maps used to initialize the ten regions for the locaNMF decomposition. Penalties within the boundaries of each region were 0 and increased exponentially with distance from the boundary. **c**, Total variance explained as a function of the number of components for locaNMF and standard singular-value decomposition (SVD) averaged across animals (n = 7, line for mean and shaded area for its s.e.). **d**, Blue = relative variance explained with respect to V1 for each of the areas ordered by their variance explained. Red = surface areas relative to V1. **e**, Fraction of the total variance explained by the locaNMF components from each region normalized by the number of pixels in each region. **f**, Number of components required in each area to reach 99% of total EV, averaged across animals. This number does not simply reflect area sizes; for instance, L decomposition resulted in many components in all animals despite being a small region. **g**, Average number of components as in **f**, normalized by the size of each region. **e-h** Error bars denote mean and its s.e. across animals (n = 7 animals).

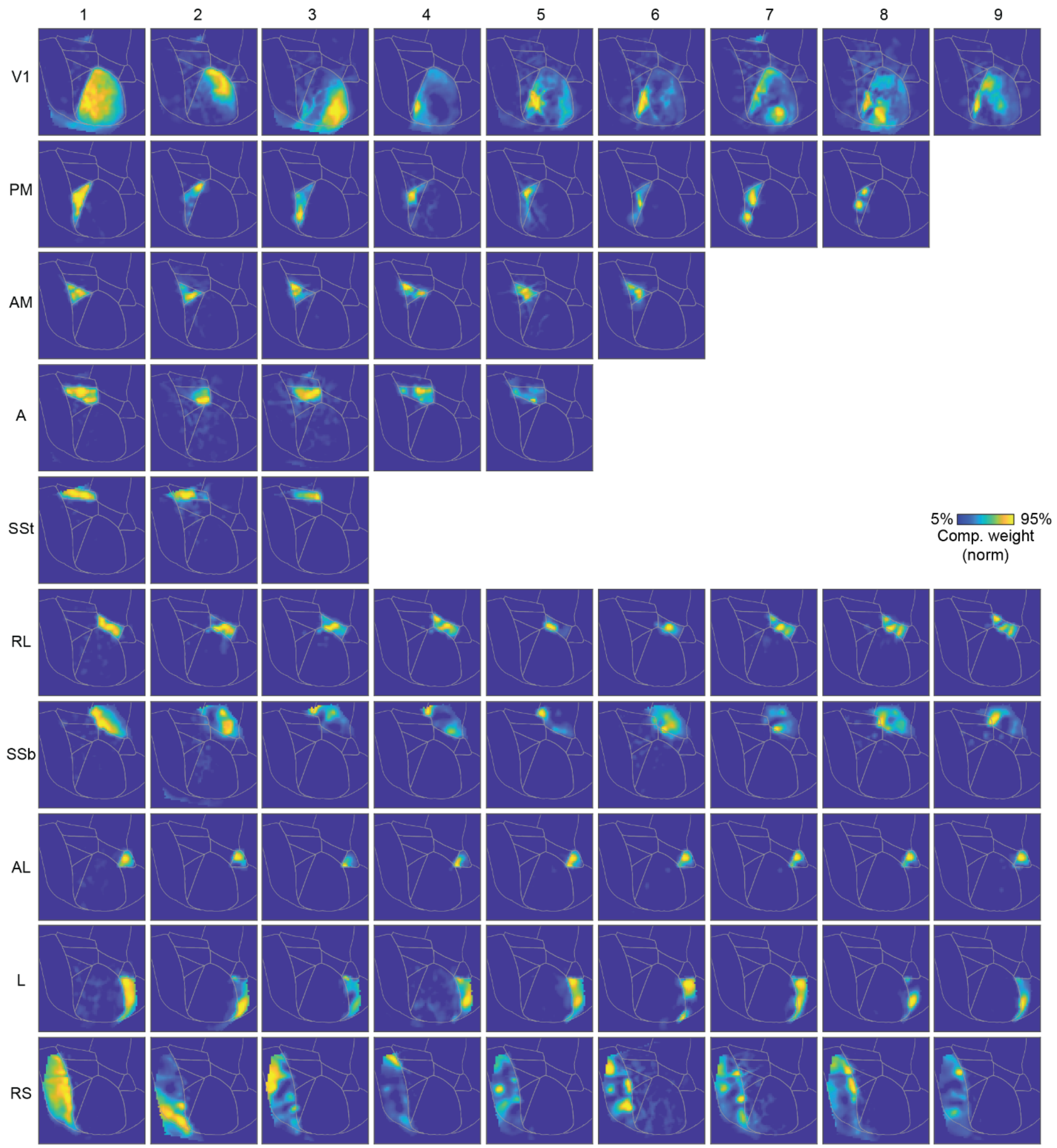

Supplementary Figure 2 | Example locaNMF for a given animal showing the top 9 components across each area. Each component color code is independently normalized relative to its maximum pixel-weight.

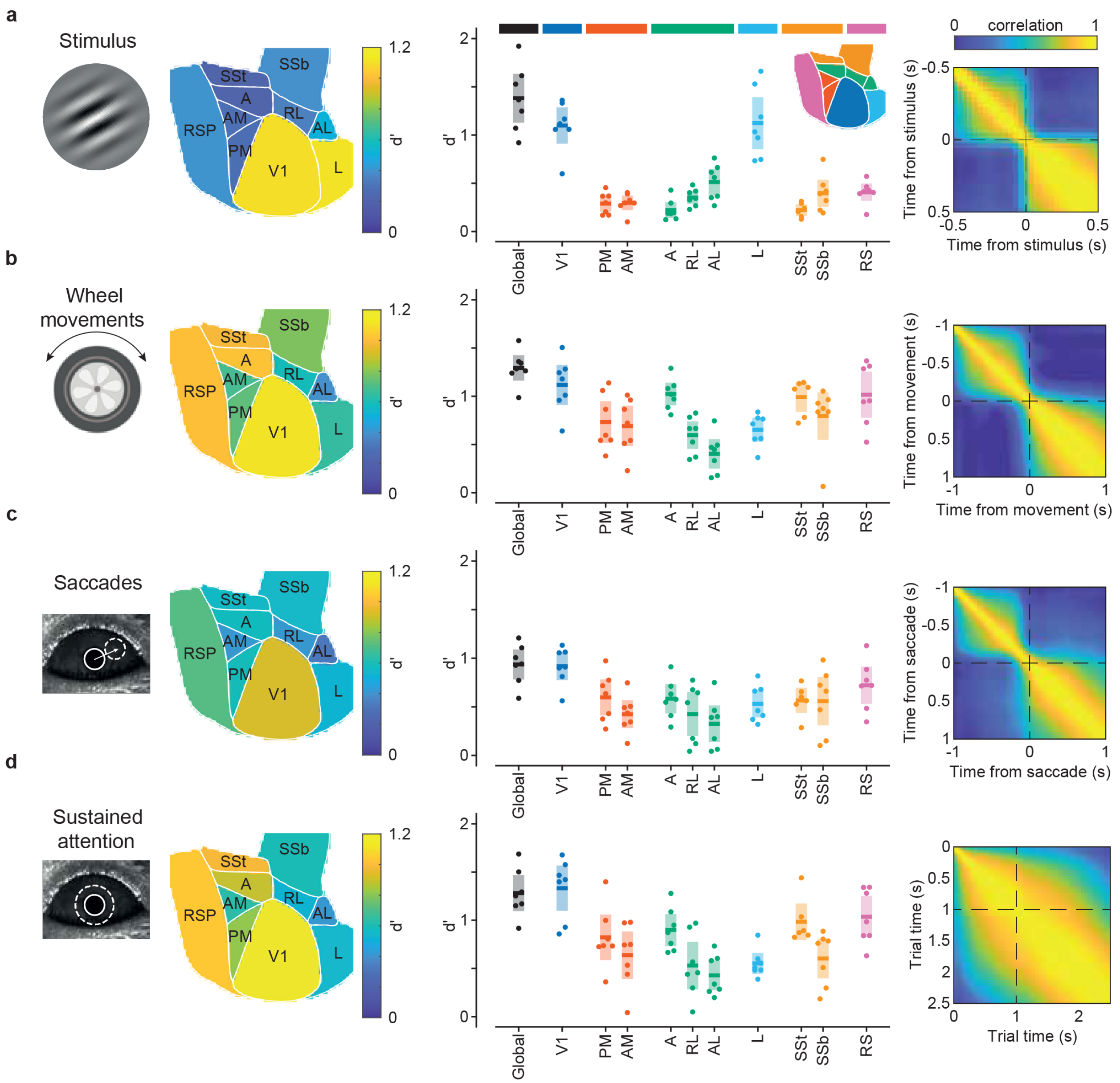

Supplementary Figure 3 | a, Average stimulus discriminability d’ for each area. Middle: Statistics for d’ values considering all areas together (global), and for each individual area across animals. Dots are different animals; middle lines and shaded areas are means and their 95% CI (n = 7 animals). Inset: color-code reference for each of the areas. Right**:** Stability of the stimulus state axis in time for a representative animal. **b-d**, same as in **a**, for wheel movements, saccades, and sustained attention.

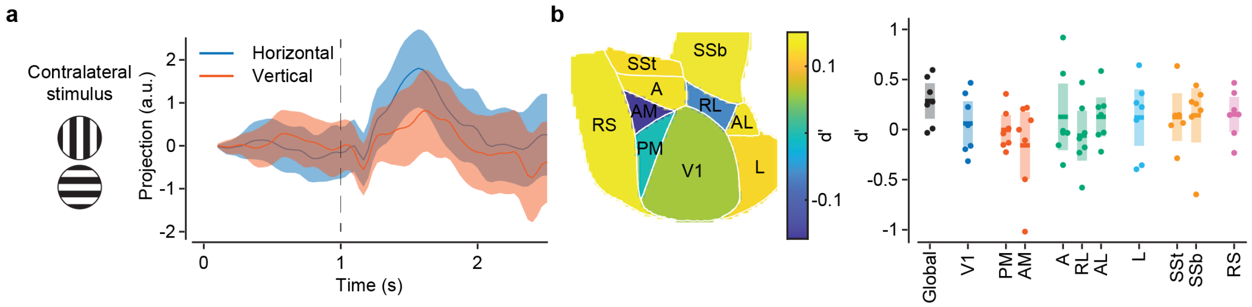

Supplementary Figure 4 | a, Widefield activity contained no information about the contralateral stimulus orientation. Time dependence of response projections onto a state axis defined using horizontal or vertical contralateral stimulus trials for a representative animal. Trajectories did not split during the trial. The line is the trial average, and shaded areas are its 95% CI for a representative animal. **b**, Statistics for global and area-specific d’ for the same state-vector. All area-based d’ values were consistent with no discriminability power. Dots are different animals; middle lines and shaded areas are means and their 95% CI (n = 7 animals).

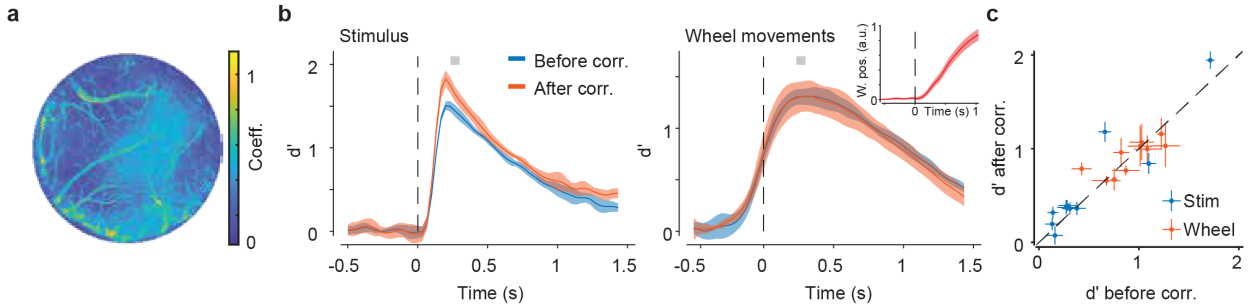

Supplementary Figure 5 | a, Hemodynamic correction. Hemodynamic correction had limited impact on state axes definitions and discriminability values. Spatial map of linear coefficients of the hemodynamic correction fit (see Methods) for a given animal. Coefficients only became substantial (> 0.5) on top of the largest blood vessels. **b**, Comparison of d' values for stimulus (left) and wheel (right) movement detection (line for trial average and shaded area for its 95% CI). Hemodynamic correction slightly increased d' values for stimulus detection at stimulus onset time, but the overall curves for wheel movements remained unchanged (Inset: Wheel position profile for the wheel movements used in the figure). **c**, Scatter plot of d' values for each of the 10 cortical areas before and after hemodynamic correction for stimulus and wheel movements state axes. For most of the areas, all pairs fell at the diagonal, denoting similar d' scores (n = 10 retinotopic areas). Linear correlation values between hemodynamic corrected and uncorrected d' values were 0.93 and 0.79 for stimulus and wheel movements respectively). Each point shows the average d' and the error bar its standard deviation across the 5 cross-validation folds.

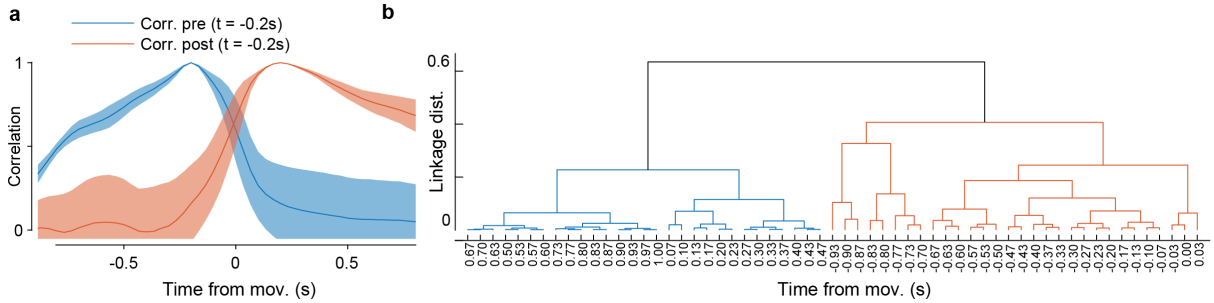

Supplementary Figure 6 | a, Cross-section of the cross-correlogram from Supplementary Fig. 2b for pre- and post-movement state axes, defined at ~200 ms before and after movement onset, i.e., at times when correlation values r = 1 in the blue and orange curve, respectively. Correlation values remain large within the pre- and post-movement periods, with correlations quickly dropping to near zero values in the “other” time interval (positive times for the blue line, and negative for the orange; lines and shaded regions are mean and s.e. of the cross-correlogram averaged across n = 7 animals). **b**, Hierarchical clustering (average linkage with correlation metric) of the movement state axis defined in pre- and post-movement time intervals for a given animal. State axes cluster in two large groups corresponding to pre- and post-movement onset (marked by the sign change). Only the first two frames post movement belong to the pre-movement cluster, and this is due to the moving window used to average the data when defining the state vectors (Methods).

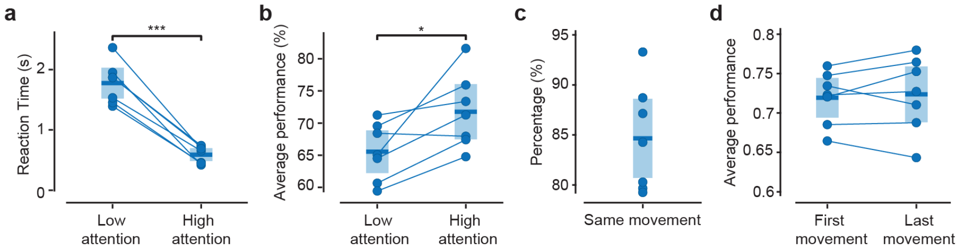

Supplementary Figure 7 | a, Reaction times for the first movement depended on attention, being significantly shorter in high attention trials (paired two-sided *t*-test, $p = 4 \times 10^{-5}$). **b**, Average performance was consistently higher in high-attention trials (paired two-sided *t*-test, p = 0.02). **c**, Fraction of occurrences when the direction of the first wheel movement coincided with the direction of the last movement in the trial (i.e., the movement that ended the trial). **d**, Comparison of overall performance when considering either the first or the last movement (paired two-sided *t*-test, p = 0.8). In all panels, a dot indicates one animal; the middle bar and shaded area are the average across animals and 95% CI. In all panels, a dot indicates one animal; the middle bar and shaded area are the average across animals and 95% CI (n = 7 animals). Lines join data from the same animal.

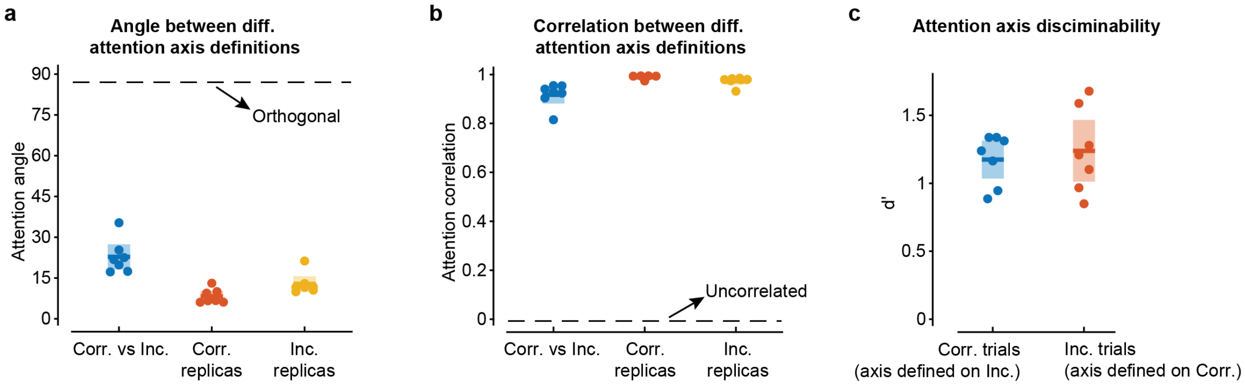

Supplementary Figure 8 | a, Angle between the state axes for sustained attention defined using either correct or incorrect trials. The angle resulting from the same state axes defined across different replicas (folds in the cross-validation procedure) are also shown for comparison for both correct and incorrect definitions. Dashed line: average angle for orthogonal axes (85.5°, obtained by resampling the components in one of the axes). **b**, Pearson correlation values for the same state axes comparisons done in **a.** Dashed line: average angle for uncorrelated axes (r = 0.02, obtained by resampling the components in one of the axes). **c,** Discriminability values obtained when discriminating attention states from correct trials using their projections onto the state axis defined with incorrect trials and vice versa. High discriminability values are maintained in both cases. In all panels, a dot indicates one animal; the middle bar and shaded area are the average across animals and 95% CI (n = 7 animals).

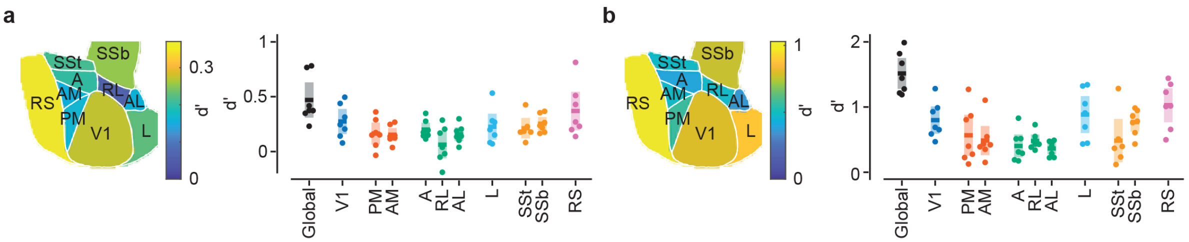

Supplementary Figure 9 | a, Area-specific d’ contributions to choice before movement onset (t = -0.1 s). Global and area-based d’ statistics for the choice state axis according to movement time (Figure 3 main text). **b**, As in (a), but after movement onset (t = 0.3 s). In all panels, a dot indicates one animal; the middle bar and shaded area are the average across animals and 95% CI (n = 7 animals).

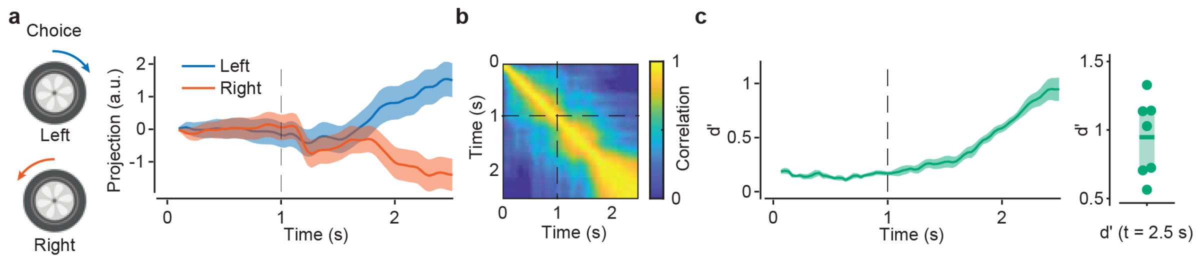

Supplementary Figure 10 | a, Choice signals can be distinguished in trial time. Projections onto the state axis for choice defined according to trial-time—not movement time— for a representative animal. Only trials in which the first movement appeared in the 1.5 to 2.5 s window were included. Trajectories started to split within the same window (line for trial average and shaded area for its 95% CI). **b**, Stability of the choice state axis in trial time. A first signature of stability appears soon after stimulus onset. **c**, Global d' evolution for the same state axis averaged across animals (left, n = 7, line for mean and shaded area for its s.e.), and statistics of peak values (right; each dot is one animal, middle bar and shaded area are the average across animals and 95% CI n = 7 animals). d' starts to increase immediately after stimulus onset and before movement onset.

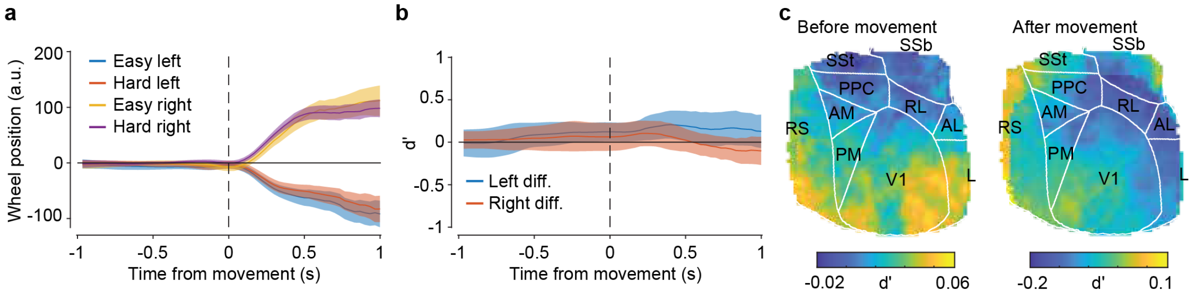

Supplementary Figure 11 | a, Choice-related wheel movements were independent of difficulty. Wheel position trajectories, aligned to movement time, separated for easy (< 30 deg) and hard (> 60 deg) trials for left and right choices for a representative animal (line for trial average and shaded area for its 95% CI). **b**, d’ discriminability values between easy and hard wheel position trajectories for left and right choices. The d’ values stayed near 0, indicating no large differences between wheel position trajectories with difficulty (n = 7, line for mean and shaded area for its s.e.). **c**, pixel-wise choice decoding. Choice d’ values computed with the activity of each pixel (local) in cortical space before (t = -0.1 s) and after (t = 0.1 s) movement onset. The d’ values were substantially lower than those obtained from locaNMF components and state axes projections. None of the pixel d’ values before movement differed significantly from baseline (p = 0.05 used as threshold, two-sided *t*-test without multiple comparisons correction, smallest p-value = 0.48).

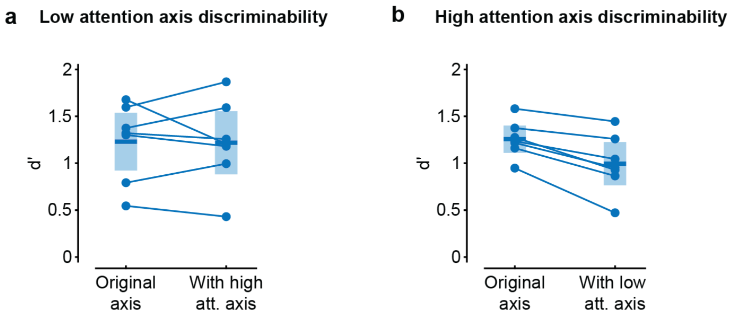

Supplementary Figure 12 | a, Stability of choice axis with attention. Left: discriminability of choice in low attention trials using the state axis defined in low attention states. Right: using the axis defined on high attention state trials instead. **b**, Same as in (a), but discriminating high attention trials instead. In all panels, a dot indicates one animal; the middle bar and shaded area are the average across animals and 95% CI (n = 7 animals).

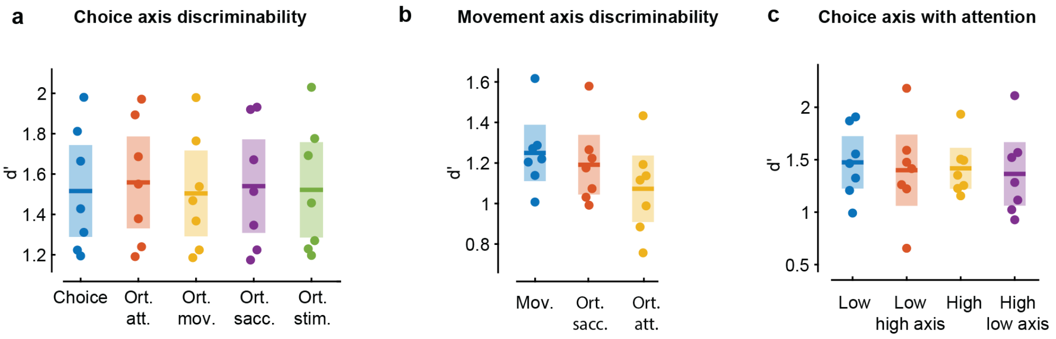

Supplementary Figure 13 | a, Stability of choice axis discriminability with other task and behavior variables. Leftmost: discriminability of the original choice axis (as in Fig. 3 main manuscript). Next: discriminability with a new axis enforced to be orthogonal to attention, movement, saccades, and stimulus axis respectively. **b**, As in **a,** but for the movement axis. In all panels, a dot indicates one animal; the middle bar and shaded area are the average across animals and 95% CI (n = 7 animals).

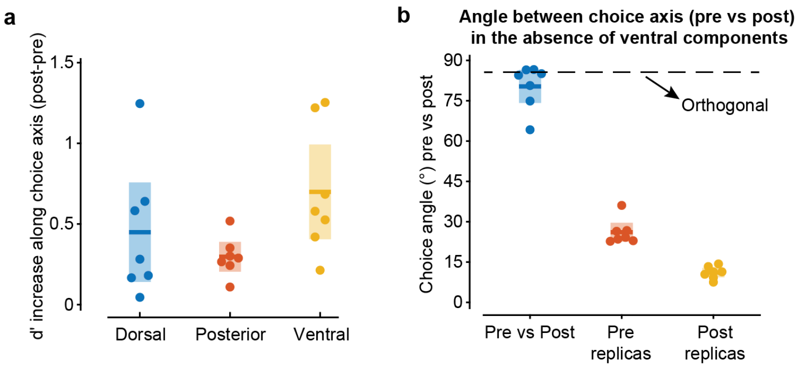

Supplementary Figure 14 | a, Average d' increase in choice discriminability from pre-movement to post-movement axes along the higher visual areas. d' increase in the ventral areas ranked significantly as the largest (5 out of 7 animals) at the 95% CI level. Rank significance was computed from 10,000 bootstrapped samples of the same d' increase-values, randomly permuted across the areas. **b,** Angle between the state axes for choice (pre- and post-movement) defined without any of the locaNMF components from area L (ventral areas). The angle resulting from the same state axes defined across different replicas (folds in the cross-validation procedure) are also shown for comparison for both pre- and post-movement axes. Dashed line: average angle for orthogonal axes (85.5°, obtained by resampling the components in one of the axes). In all panels, a dot indicates one animal; the middle bar and shaded area are the average across animals and 95% CI (n = 7 animals).

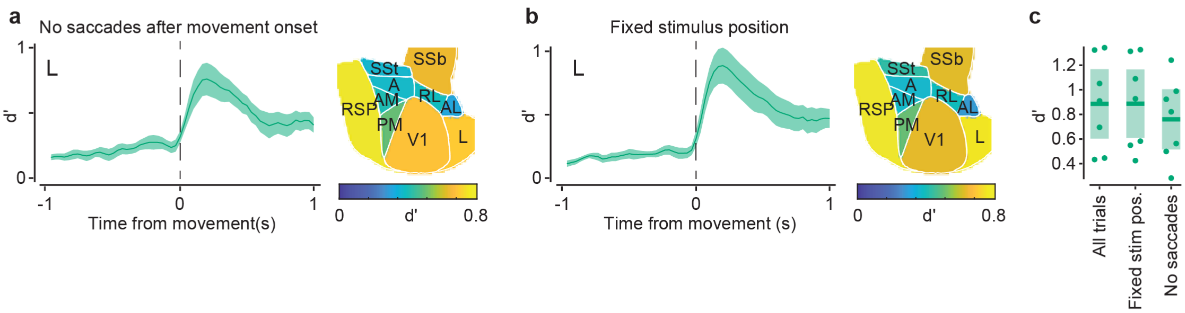

Supplementary Figure 15 | a, Ventral stream choice signature is not linked to eye or stimulus movements. Evolution of choice d' for area L during trials where the first movement occurred within 1 s of the stimulus presentation, i.e., the stimulus was always in the same position on the screen. Right: area-specific d' 0.2 s after movement for the same trials. **b**, As in **a,** but also with the constraint that there were no saccades 0.5 s before or after the movement onset. For d' trajectories: line for mean across animals and shaded area for its s.e. (n = 7). **c**, Comparison of peak d' values in area L for the three controlled conditions: all trials (same as Fig. 3 on the main text), and those shown here in panels **a**, and **b**. In all panels, a dot indicates one animal; the middle bar and shaded area are the average across animals and 95% CI (n = 7 animals).

			Task related				Choice related			
	Reference	Type	Memory	Evidence	Regions & resolution	Choice assesment	Causality	Choice regions	Species	Notes
1	Zatka-Haas et al., bioRxiv 2021	HF, 2AFC VIS contrast discrimination	✗	St	Neuropixel + whole cortex widefield	Binary decoder, pre-motor sensitivity	✓	Rare, mostly in MOs	MOU	Choice as action selection; perhaps task driven by basal ganglia, SC, and zona incerta
2	Orsolic et al., Neuron 2021	HF, go-nogo, speed change VIS detection task	✗	St	Whole cortex widefield, 2P in MOs	n/a, but see 'temporal expectation'	✗	n/a	MOU	MOs and MOp responded to pro-licking stimulus fluctuations when speed change was likely
3	Osako et al., Curr. Biol. 2021	FM, 3-choice VIS detection task + 2AFC detection	✗	St	Tetrods in PPC and V1	d-prime, distance of projections onto activity modes, SVM decoder	✗	PPC & V1	RAT	No explicit left-right choice encoding
4	Lee et al., bioRxiv 2020	HF, 2AFC VIS T-maze + wheel task	✗	St	PPC, 2P	ccCP	✗	PPC	MOU	Different motor actions for choice across tasks; choice encoding in T-maze, rarer in wheel task
5	Tang & Higley, Neuron 2020	HF, visually cued, eyeblink conditioning taks	✗	St	V1 L5, 2P	Logistic regression (prior blink)	✓	V1	MOU	CPs responses more predicitive than CSt and causally needed for performance
6	Salkoff et al., Cerebral Crtx. 2020	HF, go-nogo VIS (LED flash) task	✗	St	Whole cortex widefield, ePhys	auROC	✓	MOs	MOU	Response ↑ in visual and somatosensory cortex in pretrial times with misses
7	Puscian et al., Cell Rep. 2020	HF, visually cued, eyeblink conditioning taks	✗	St	V1, 2P	Linear classifier to predict blinks	✗	V1	MOU	Prediction accuracy stronger in late training phase, in pyramidal and PV
8	Kauvar et al., Neuron 2020	HF, 3-option lick, go-nogo history-guided odor task	✓	St	All cortex COSMOS imaging (1-15 cells)	PLS-based decoder	✓	Distrbuted, also V1	MOU	Videography predics motor action in pre-odor periods
9	Koay et al., eLife 2020	HF, VIS T-maze delayed 2AFC navigation task	✓	Pl	Visual-parietal cortex and RSC, 2P ROIs	SVM decoder on regressive model, corrected by view angle	✗	Gradients (V1 the least)	MOU	Uncorrelated mode analysis to isolate choice from motor; focus on small sample of cue-locked cells
10	Koay et al., bioRxiv 2019	HF, VIS T-maze delayed 2AFC navigation task	✓	Pl	Visual-parietal cortex and RSC, 2P ROIs	Two-sample t-test in active periods	✗	Uniformly distributed	MOU	Multiplicative neural sequences for efficient coding
11	Minderer et al., Neuron 2019	HF, locomotion with VIS optic flow in VR ("distance" task)	✗	St	Widefield, 2P, dorsal parietal cortex, RSC	Not examined	✓	n/a	MOU	Opto Inhibition at widefield level: visual, parietal, RSC
12	Musall et al., Nat. Neurosci. 2019	HF, delayed, 2AFC VIS spatial discrimination (1s delay) task, VIS or AUD	✓	Pl	Widefield whole cortex, 2P, Neuropixel	Regressive model, not pre-motor sensitive	✗	ALM(?)	MOU	Unclear which region weigthed choice the most
13	Zhong et al., Nat. Neurosci. 2019	HF, 2AFC AUD licking task	✗	St	PPC, A1	ROC, linear classifier	✓	PPC	MOU	PPC↓ affected new stims & recategorization, not familiar ones, and reduced hist. biases
14	Pinto et al., Neuron 2019	HF, three 2AFC VIS navigation tasks	✓	Pl & St	Widefield whole cortex	Pixel & area based decoder, with view-angle info	✓	Posterior to frontal gradient in decoding accuracy	MOU	Inactivation effects task complexity; distributed nets necessary for memory and accumlation tasks; only VIS-PPC in visually guided
15	Steinmetz et al., Nature 2019	HF, 2AFC VIS contrast discrimination	✗	St	Neuropixel, whole brain	Regressive model, ccCP, pre-motor sensitivity	✗	Rare, in forebrain (MOs, PL, MOp) BG, SCm, hTH	MOU	Vertical Neuropixel penetration in cortex
16	Pho et al., Nat. Comms. 2018	HF, go-nogo (licking) VIS task	✗	St	V1, PPC, 2P	Time-dependent ROC, FA vs CR	✓	PPC	MOU	Reversed sensorymotor contingency
17	Odoemene et al., J. Neurosci. 2018	FM, 2AFC VIS ligh flashes accumulation task	✗	Pt	Visual areas, widefield	n/a	✓	n/a	MOU	Early flashes larger weigths similar to mokeys but not rats; AM inactivation biases decision
18	Akrami et al., Nature 2018	FM, 2AFC AUD discrimination (louder)	✓	St	PPC, ePhys	Mutual information	✓	PPC	RAT	Performance ↑ with reduction of history bias (but ↓when bias helped)
19	Gilad et al., Neuron 2018	HF, tactile go-nogo texture discrimination	✓	St	Widefiled whole cortex & 2P in S1,2 RL, PPC	SVM Hit vs CR	✓	S1, S2, RL	MOU	Short-term memory: M2 future actions, P past stims.
20	Krumin et al., eLife 2018	HF, 2AFC VIS contrast-detection navigation task	✗	St	PPC, 2P	Local likelihood method	✗	n/a	MOU	Heading and position explains decision
21	Licata et al., J. Neurosci. 2017	HF, delayed, rate discrimination task, VIS or AUD	✗	Pl	PPC, ePhys	Regressive model for inactiovation; MI for neural	✓	PPC	RAT	PPC↓ spares auditory but not visual decisions

			Task related			Choice related				
	Reference	Type	Memory	Evidence	Regions & resolution	Choice assesment	Causality	Choice regions	Species	Notes
22	Driscoll et al., Cell 2017	HF 2AFC VIS T-maze navigation task	✓	St	PPC, 2P	GLM and C-SVC	✓	PPC	MOU	PPC↓ most effective during cue period
23	Scott et al., Neuron 2017	HF, 2AFC VIS discrimination	✓,✗	Pt	PPC, FOF, mV2, 2P, ePhys	SVM decoder	✗	FOF	RAT	Heterogeneous dynamics in response to individual pulses as a 'temporal basis' for evidence accumulation
24	Hwang et al., Nature Comms. 2017	2AFC VIS discrimination	✓	St	PPC, 2P	auROC, linear classifier	✓	PPC	MOU	Action-selction history bias in PPC; PPC↓ no effects after stim-on
25	Chen et al., Neuron 2017	2AFC tactile licking task	✓	St	ALM, MM, 2P, ePhys	Regressive model	✗	ALM	MOU	Directional activity 1st in deep ALM, secs before movement
26	Allen et al., Neuron 2017	HF, olfactory go-nogo	✗	St	Whole cortex multi-ROI 2P	ROC + GLM, not pre-motor sensitive	✓	Unspecific cortex-wide	MOU	M2↓ no global activity ramps; single regions are necessary for global patterns (behavior)
27	Yang et al., Nat. Neurosci. 2016	HF, go-nogo tactile detection task	✗	St	2P and intracellular in S1, recording in thalamus, S2	Detection probability, not pre-motor sensitive	✗	S1	MOU	Choice carried by top-down axons from secondary somatosensory cortex
28	Kwon et al., Nat. Neurosci. 2016	HF, go-nogo tactile detection task	✗	St	2P, S1 & S2	auROC	✓	S2 (&S1)	MOU	S2 more associated to perceptual outcome than S1
29	Goard et al., eLife 2016	HF, VIS go-nogo licking VIS task	✓	St	V1, PPC, fMC, 2P	auROC and regression	✓	PPC fMC	MOU	PPC↓ in response period or delay after stimulus does not affect behavior (fMC↓ does - memory)
30	Morcos & Harvey, Nat. Neurosci. 2016	HF, 2AFC VIS T-maze navigation	✗	Pl	PPC, 2P	SVM	✗	PPC	MOU	
31	Funamizu et al., Nat. Neurosci. 2016	HF, auditory VR navigation (reach goal location)	✓	St & Pl	PPC, PM, 2P	t-test and linear regression	✓	PPC	MOU	Interpreting "goal aligned" as choice related
32	Poort et al., Neuron 2015	HF, go-nogo VIS & olfactory discrimination with navigation	✗	St	V1, 2P	Cumulative decoder	✓	V1	MOU	Choice in cells tuned to rewarded stimuli (reward expectation?)
33	Erlich et al., eLife 2015	FM, 2AFC AUD task	✗	Pl	PPC, FOF, Behavior	Behavioral	✓	FOF, PPC	RAT	PPC↓ no effect on choice driven by sound, but impairs 'internal' decisions
34	Hanks et al., Nature 2015	FM, 2AFC AUD discrimination	✗	Pl	PPC, FOF, ePhys	ROC	✓	FOF	RAT	FOF↓ has effects if at stimulus end; PPC↓ no effect
35	Guo et al., Neuron 2014	HF, 2AFC pole detection task	✓	St	S1, ALM, ePhys	Spike count (t-test)	✓	ALM	MOU	Widefield opto-inactivation
36	Raposo et al., Nat. Neurosci. 2014	FM, 2AFC VIS and AUD clicks	✗	Pl & St	PPC, ePhys	ROC, choice divergence and preference; SVM decoder	✓	PPC	RAT	All-session inactivation affected VIS not AUD
37	Harvey et al., Nature 2012	HF, VIS T-maze navigation	✓	St	PPC, 2P, ePhys	Trajectory selectivity index	✓	PPC	MOU	PPC↓ affecs memory guided task
38	Erlich et al., Neuron 2011	FM, 2AFC AUD	✓	St	FOF, ePhys	ROC	✓	FOF	MOU	Head angle did not explain FOF delay period rates predicting orienting choice
39	Jaramillo & Zador, Nat. neurosci. 2011	HF, 2AFC AUD task	✗	St	AC, ePhys	n/a, but see 'temporal expectation'	✓	AC	MOU	

St = static; Pl = pulsatile; HF = head fixed; FM = freely moving; MOU = mouse; VIS = visual; AUD = auditory; 2P = two photon imaging; ePhys = elecrophysiology; SVM = support vector machine; ROC = receiver operating characteristic; ccCP = combined conditions choice probability; GLM = generalized linear model; CR = correct rejections; C-SVC = C-support vector classification; PLS = partial least square regression; MI = mutual information; ↓↑= decreased/insreased activations.

PPC = posterior parietal cortex; V1 = primary visual cortex; S1 = primary somatosensory cortex; MO_s = secondary motor cortex (M2); MO_p = primary motor cortex (M1); ALM = anterolater motor cortex; SC = superior colliculus; SC_m = medial part of SC; RSC = retrosplenial cortex; FOF = frontal orienting field; MM = medial motor cortex; AM = anteromedial visual cortex; AC = auditory cortex; A1 = primary auditory cortex; BG = basal ganglia; hTH = high-order thalamus; P = posterolateral visual area; CPn = corticopontine; CSt = corticostriatal; fMC = frontal motor cortex; mV2 = medial secondary visual cortex; RL = rostrolateral posterior parietal cortex; PL = prelimbic area; L5 = layer 5.

Supplementary Table 1 | Summary of recent rodent studies focused on choice signals. Listed works focus primarely on the posterior cortex, with a few examining fronto-parietal or whole-cortex networks. This table reflects the authors' selection of a limited number of studies judged as particulary relevant for this work.

Supplementary Table 2 | Trial conditions and number of trials used to obtain the different state axis used throughout the text.

State axis definition (maximize discriminability between trial sets A and B)	***Trial set A (avg num of trials per animal)***	***Trial set B (avg num of trials per animal)***	***Notes***
Stimulus	Stimulus present (2730)	No stimulus present (2730)	Number of no stimulus conditions chosen to match the number of stimulus (randomly chosen)
Wheel movements	Trials with a wheel movement (left or right) after the stimulus, without any other movements before the stimulus and without any saccades within 1s of the movement (1190)	Trials without a wheel movement (left or right) after the stimulus, without any other movements before the stimulus and without any saccades within 1s of the movement (1190)	Number of no wheel conditions chosen to match the number of stimulus (randomly chosen)
Saccades	Trials with a saccade after the stimulus, without any other saccades before the stimulus and without any wheel movements within 1s of the movement (465)	Trials without a saccade after the stimulus, without any other saccades before the stimulus and without any wheel movements within 1s of the movement (465)	Number of no saccade conditions chosen to match the number of stimulus (randomly chosen)
Sustained attention	Top 33rd percentile of pupil area change trials (896)	Bottom 33rd percentile of pupil area change trials (896).	
Choice	Trials with a right choice during the closed loop (657)	Trials with a left choice during the closed loop (721)	Pre- and post- movement axes were defined at different time points within the trial (before and after movement onset)
Attention on Corr. Trials	Top 33rd percentile of pupil area change trials that resulted in correct choices (668)	Bottom 33rd percentile of pupil area change trials that resulted in correct choices (592)	
Attention on Incor. Trials	Top 33rd percentile of pupil area change trials that resulted in incorrect choices (221)	Bottom 33rd percentile of pupil area change trials that resulted in incorrect choices (248)	
Choice with high attention and easy trials	Trials where the animal made a right choice during the closed loop on the top 33rd percentile of pupil area change and with angle difference > 45 deg (142)	Trials where the animal made a left choice during the closed loop on the top 33rd percentile of pupil area change and with angle difference > 45 deg (127)	
Difficulty with high attention and hard trials	Trials where the animal made a right choice during the closed loop on the top 33rd percentile of pupil area change and with angle difference < 45 deg (177)	Trials where the animal made a left choice during the closed loop on the top 33rd percentile of pupil area change and with angle difference < 45 deg (206)	
Contralateral stimulus information	Left stimulus horizontal, angle difference = 90 deg and left choice (70)	Left stimulus vertical, angle difference = 90 deg and left choice (70)	
