# Supplementary material for: Distributed context-dependent choice information in mouse posterior cortex
Source: Nat Commun. 2023 Jan 12;14:192. doi: 10.1038/s41467-023-35824-6 (PMC9837177; doi:10.1038/s41467-023-35824-6)
Supplement: Supplementary file 3 — Reporting Summary [file 41467_2023_35824_MOESM3_ESM.pdf]

nature portfolio

Corresponding author(s): Andrea Benucci

Last updated by author(s): Dec 15, 2022

Reporting Summary

Nature Portfolio wishes to improve the reproducibility of the work that we publish. This form provides structure for consistency and transparency in reporting. For further information on Nature Portfolio policies, see our Editorial Policies and the Editorial Policy Checklist.

Statistics

For all statistical analyses, confirm that the following items are present in the figure legend, table legend, main text, or Methods section.

n/a	Confirmed	
☐	☒	The exact sample size (*n*) for each experimental group/condition, given as a discrete number and unit of measurement
☐	☒	A statement on whether measurements were taken from distinct samples or whether the same sample was measured repeatedly
☐	☒	The statistical test(s) used AND whether they are one- or two-sided<br>*Only common tests should be described solely by name; describe more complex techniques in the Methods section.*
☒	☐	A description of all covariates tested
☐	☒	A description of any assumptions or corrections, such as tests of normality and adjustment for multiple comparisons
☐	☒	A full description of the statistical parameters including central tendency (e.g. means) or other basic estimates (e.g. regression coefficient) AND variation (e.g. standard deviation) or associated estimates of uncertainty (e.g. confidence intervals)
☐	☒	For null hypothesis testing, the test statistic (e.g. *F*, *t*, *r*) with confidence intervals, effect sizes, degrees of freedom and *P* value noted<br>*Give P values as exact values whenever suitable.*
☒	☐	For Bayesian analysis, information on the choice of priors and Markov chain Monte Carlo settings
☒	☐	For hierarchical and complex designs, identification of the appropriate level for tests and full reporting of outcomes
☐	☒	Estimates of effect sizes (e.g. Cohen's *d*, Pearson's *r*), indicating how they were calculated

Our web collection on statistics for biologists contains articles on many of the points above.

Software and code

Policy information about availability of computer code

Data collection	Imaging data was collected using custom software based on PCO SDK (v 1.14), eye-tracking data was collected using custom software based on FlyCapture2 SDK (v8.0), with custom code written in MATLAB (r2020a) to interface with both acquisition systems using MATLAB Data Acquisition Toolbox for National Instruments (NI) devices. Acquisition software is available upon reasonable request.
Data analysis	Code for data processing, analysis and figures is available through ZENODO (CBS-NCB/distributedDM: Public release (v1.1). Zenodo. https://doi.org/10.5281/zenodo.7435887). This code was written in MATLAB (r2020a) and Python (v3.7) with Tensorflow 2.0.

For manuscripts utilizing custom algorithms or software that are central to the research but not yet described in published literature, software must be made available to editors and reviewers. We strongly encourage code deposition in a community repository (e.g. GitHub). See the Nature Portfolio guidelines for submitting code & software for further information.

Data

Policy information about availability of data

All manuscripts must include a data availability statement. This statement should provide the following information, where applicable:

- Accession codes, unique identifiers, or web links for publicly available datasets
- A description of any restrictions on data availability
- For clinical datasets or third party data, please ensure that the statement adheres to our policy

Source data are provided with this paper. Data required to replicate the findings is available through ZENODO (CBS-NCB/distributedDM: Public release (v1.1). Zenodo. https://doi.org/10.5281/zenodo.7435887). Unprocessed raw data is too large for permanent external storage and will be made available by the

corresponding author upon reasonable request. Atlas reference data for brain areas alignment across animal is obtained from The Allen Mouse Common Coordinate Framework (CCF) (https://atlas.brain-map.org/)

Human research participants

Policy information about studies involving human research participants and Sex and Gender in Research.

Reporting on sex and gender	Not applicable
Population characteristics	Not applicable
Recruitment	Not applicable
Ethics oversight	Not applicable

Note that full information on the approval of the study protocol must also be provided in the manuscript.

Field-specific reporting

Please select the one below that is the best fit for your research. If you are not sure, read the appropriate sections before making your selection.

[x] Life sciences [ ] Behavioural & social sciences [ ] Ecological, evolutionary & environmental sciences

For a reference copy of the document with all sections, see nature.com/documents/nr-reporting-summary-flat.pdf

Life sciences study design

All studies must disclose on these points even when the disclosure is negative.

Sample size	Sample size was established based on the need to collect data within a reasonable time-frame and in adherence to standards in the field. Seven adult mice were used for the experiments presented in this study.
Data exclusions	Experimental sessions in which animals were mostly disengaged form the behavioral task were excluded. All additional exclusions are described in the Methods section in detail.
Replication	Data was colleced across seven different animals (independently collected). No other attemps to replication were made.
Randomization	Not applicable. We considered a single experimental condition.
Blinding	Not applicable. We considered a single experimental condition.

Reporting for specific materials, systems and methods

We require information from authors about some types of materials, experimental systems and methods used in many studies. Here, indicate whether each material, system or method listed is relevant to your study. If you are not sure if a list item applies to your research, read the appropriate section before selecting a response.

Materials & experimental systems

n/a	Involved in the study	
[x]	[ ]	Antibodies
[x]	[ ]	Eukaryotic cell lines
[x]	[ ]	Palaeontology and archaeology
[ ]	[x]	Animals and other organisms
[x]	[ ]	Clinical data
[x]	[ ]	Dual use research of concern

Methods

n/a	Involved in the study	
[x]	[ ]	ChIP-seq
[x]	[ ]	Flow cytometry
[x]	[ ]	MRI-based neuroimaging

Animals and other research organisms

Policy information about studies involving animals; ARRIVE guidelines recommended for reporting animal research, and Sex and Gender in Research

Laboratory animals	Mus musculus; Thy1-GCaMP6f (n=7, 6 males and 1 female); ages 5-12 months; Housing was done using a 12 light/12 dark cycle, with

	~18-23°C temperature and with 40-60% humidity
Wild animals	The study did not involve wild animals
Reporting on sex	Data has not been disaggregated based on sex in addherence with current standards in the field.
Field-collected samples	No field collected samples were used in the study.
Ethics oversight	All procedures were reviewed and approved under the institutional Animal Care and Use Committee of RIKEN (protocol number: W2019-131215).

Note that full information on the approval of the study protocol must also be provided in the manuscript.
